# Mangiferin suppresses human metastatic osteosarcoma cell growth by down-regulating the expression of metalloproteinases-1/2 and parathyroid hormone receptor 1

**DOI:** 10.1186/s13568-020-0949-4

**Published:** 2020-01-18

**Authors:** Jifeng Wen, Yong Qin, Chao Li, Xiankui Dai, Tong Wu, Wenzhe Yin

**Affiliations:** 1 Department of Gastroenterology, The Second Affiliated Hospital of Harbin Medical University, Harbin, China; 2 Department of Orthopedics, The Second Affiliated Hospital of Harbin Medical University, Xuefu Road 246, Nangang District, Harbin, Heilongjiang China

**Keywords:** Mangiferin, Proliferation, Adhesion, Osteosarcoma, mRNA

## Abstract

The study evaluates the protective effect of mangiferin on osteosarcoma cell proliferation and metastasis. Saos-2 and U2OS cells were treated with mangiferin (25, 50, 75 and 100 µM) for 72 h. Mangiferin reduced the cell viability, invasion, and cell adhesion and migration rate. Matrix metalloproteinases-2/9 (MMP-2/9) mRNA expression was reduced significantly, while the levels of tissue inhibitors of metalloproteinases-1/2 (TIMP-1/2) were elevated in Saos-2 and U2OS cells. Mangiferin treatment significantly reduced parathyroid hormone receptor 1 (PTHR1) mRNA and protein expression by more than 0.5-fold in both osteosarcoma cells. In addition, the immunofluorescent analysis also showed decreased PTHR1 expression following treatment with mangiferin. In summary, we have demonstrated that treatment with mangiferin reduces cell viability, proliferation, invasion, adhesion and migration, and induces apoptosis of osteosarcoma cells. Therefore, treatment with mangiferin can be effective agent in inhibiting growth and inducing apoptosis in osteosarcoma cells. Our experimental results provide evidence for the therapeutic effect of mangiferin in osteosarcoma cells.

## Introduction

Osteosarcoma is severe malignant bone tumor (Li et al. 2018), and teenagers and adults are affected mostly by osteosarcoma (Luetke et al. 2014). Although water fluoridation is believed to be the main cause of osteosarcoma without clear research data to conclude this (Iida and Kumar 2009). Ottaviani and Jaffe (2009) have reported the increased mortality rate in childhood due to malignant bone and joint tumor. Chemotherapy and surgical resection have improved the survival rate of osteosarcoma up to 70%. However, osteosarcoma recurrence and metastasis leads to an increased mortality rate (Moreno et al. 2017; Berner et al. 2015). Parathyroid hormone receptor 1 (PTHR1) plays a major role in the pathophysiology of osteosarcoma (Lupp et al. 2010) and expressed in metastatic cells and tissues (Ho et al. 2014). Ho et al. (2015) have reported that the PTHR1 knockdown in osteosarcoma cells decreases the growth and invasion, and enhances tumor differentiation. Overexpression of PTHR1 in osteosarcoma increases motility and proliferation. Furthermore, it delays upregulation of genes which are responsible for the extra cellular matrix (ECM) production and osteoblastic differentiation (Ho et al. 2015). The putative mechanism suggested for PTHR1 is parathyroid hormone (PTH) is known to downregulate the expression of PTHrP receptor in osteoblast-like cells through a cAMP-dependent and PKA-independent pathway (Kawane et al. 2003).

Mangiferin is well-known xanthone found in several mango fruits such as barks, peel, leaves, stone, stalks and kernel, and in higher plants (Imran et al. 2017). Dar et al. (2005) have reported the several pharmacological effects of mangiferin such as antioxidant, anticancer, antiaging, antiviral, hepatoprotective, analgesic and immunomodulatory potential. Thus, the study analyzed the ability of mangiferin suppresses human metastatic osteosarcoma cell growth by down-regulating the expression of MMP-2/9 and PTHR1.

## Materials and methods

Mangiferin was purchased from the Supelco Inc. (06279, Pennsylvania, USA). Chondro T, DMEM, penicillin–streptomycin and FBS were obtained from Sigma-Aldrich (Shanghai, China). Anti-human IgG-H&L (fluorescein isothiocyanate; FITC), PTHR1 antibody (SAB5300029), and an apoptosis kit (APOAF-20TST), trypsin–EDTA and antibiotics were also purchased from Sigma-Aldrich.

### Cell culture

Saos-2 and U2OS cells were obtained from ATCC to cultured in M199 medium containing heparin, antibiotics (1%) and FBS (10%) at room temperature with 5% CO_2_. The preliminary investigation was carried out with different concentration of mangiferin from 25 to 200 µM. However, we noted the optimum and significant effect between 25 and 100 µM of mangiferin. Thus, we selected these concentrations in this study.

### Cell viability assays

Saos-2 and U2OS cells were seeded (1.5 × 10^4^ cells/well) in growth medium treated with mangiferin (25, 50, 75 and 100 µM) for 72 h. Then, cells were incubated with sulfordhamine-B (SRB) solution for the calculation of osteosarcoma cell viability and inhibition (Pandurangan et al. 2017).

### Clonogenic assays

Saos-2 and U2OS cells were seeded (1.1 × 10^4^ cells/well) in growth medium treated with mangiferin (25, 50, 75 and 100 µM) for 72 h and crystal violet (0.2%) was used for staining for 30 min at 37 °C. Cells were washed with water and the cell survival rate was compared to the appropriate controls (Chaudhary et al. 2015).

### Annexin-V/PI staining

Saos-2 and U2OS cells were seeded (1.5 × 10^4^ cells/well) in growth medium and incubated with mangiferin (50, 75 and 100 µM) for 72 h, and stained with the Annexin-V to visualize apoptotic cells and propidium iodide (PI) to identify necrotic cells after repeated washing with phosphate-buffered saline (PBS) (Kramer et al. 2013).

### Determination of cell detachment

Saos-2 and U2OS cells were seeded (1.5 × 10^4^ cells/well) in growth medium and treated with mangiferin (25, 50, 75 and 100 µM) for 72 h and detached cell was determined by flow cytometer (CytoFLEX LX, Beckman Coulter, Indiana, USA).

### Cell adhesion assays

Saos-2 and U2OS cells were seeded (1.5 × 10^4^ cells/well) and treated with mangiferin (25, 50, 75 and 100 µM) for 72 h, washed repeatedly with PBS, and stained with hematoxylin and eosin (H&E). The stained image was analyzed under a microscope and the cell adhesion rate was calculated (Do Thi and Hwang 2014).

### Cell invasion assays

Saos-2 and U2OS cells were seeded (1.5 × 10^4^ cells/well) in growth medium and treated with mangiferin (25, 50, 75 and 100 µM) for 72 h and percent cell invasion was determined as described previously (Do Thi and Hwang 2014).

### Wound healing migration assays

Saos-2 and U2OS cells were seeded (1.5 × 10^4^ cells/well) in growth medium and treated with mangiferin (25, 50, 75 and 100 µM) for 72 h and the percent wound healing (migration) was determined as reported previously **(**Justus et al. 2014).

### Rt-PCR

Saos-2 and U2OS cells were seeded (2.5 × 10^4^ cells/well) in growth medium and treated with mangiferin (25, 50, 75 and 100 µM) for 72 h and RNA was extracted and converted into cDNA. Then, qPCR was performed for matrix metalloproteinases-2/9 (MMP-2/9), tissue inhibitors of metalloproteinases-1/2 (TIMP-1/2), PTHR1, and glyceraldehyde 3-phosphate dehydrogenase (GAPDH) (Table 1). The mRNA expression was determined (Muthuraman et al. 2014).

**Table 1:** List of RT-PCR primers used in this study

S.no	Gene name	Forward primer	Reverse primer
1	PTHR1	5′-CTCTTTGGCGTCCACTACATTG-3′	5′-TTGAGGAACCCATCGTCCTTG-3′
2	MMP-2	5′-GGC CCT GTC ACT CCT GAG AT-3′	5′-GGC ATC CAG GTT ATC GGG GA-3′
3	MMP-9	5′-CGG AGC ACG GAG ACG GGT AT-3′	5′-TGA AGG GGA AGA CGC ACA GC-3′
4	TIMP-1	5′-GTT CAA CAC CTC CAT GTT GGT GGA C-3′	5′-TGG TGT TGA GGC AGA CCA GCT TC-3′
5	TIMP-2	5′-GGT CTG GTG CCT GGT CTG ATG ATG-3′	5′-GTC CTT TCA AGG AGA ATG GTC G-3′
6	GAPDH	5′-GGTCACCAGGGCTGCTTTT-3′	5′-ATCTCGCTCCTGGAAGATGGT-3′

### Western blot analysis

Western blot experiment was used to measure the cellular effect of mangiferin on PTHR1 protein expression. Saos-2 and U2OS cells were seeded (2.5 × 10^4^ cells/well) in growth medium and incubated with mangiferin (50, 75 and 100 µM) for 72 h. Protease inhibitor was used to lyse the cells in cell lysis buffer, and cell extract was collected by centrifugation and protein content in the extract was determined by Bradford assay. Proteins in the extract were separated membrane, and non-fat (5%) was incubated with the immunoblots to block non-specific sites and then incubated with primary antibodies (1:300 dilution ratio) overnight at cold temperature. Then, blots were incubated with HRP-conjugated antibodies for 60 min at cold temperature. The protein levels were visualized by Dmitriev et al. (2005).

### Immunofluorescence

Saos-2 and U2OS cells were seeded (2 × 10^4^ cells/well) in growth medium and treated with mangiferin (75 and 100 µM) for 72 h, Then, cell was incubated with BSA for 60 min and treated with PTHR1 antibody for 12 h and followed by fluorescein isothiocyanate (FITC)-conjugated antibody for 60 min (Balic et al. 2011).

### Statistical analysis

Results are given as mean ± standard error of the mean (SEM). Student’s *t*-test was used to compare the groups, and analysis of variance (ANOVA). The *P *< 0.05 was considered as significant.

## Results

### Effect of mangiferin on osteosarcoma cell viability

**Fig. 1:**
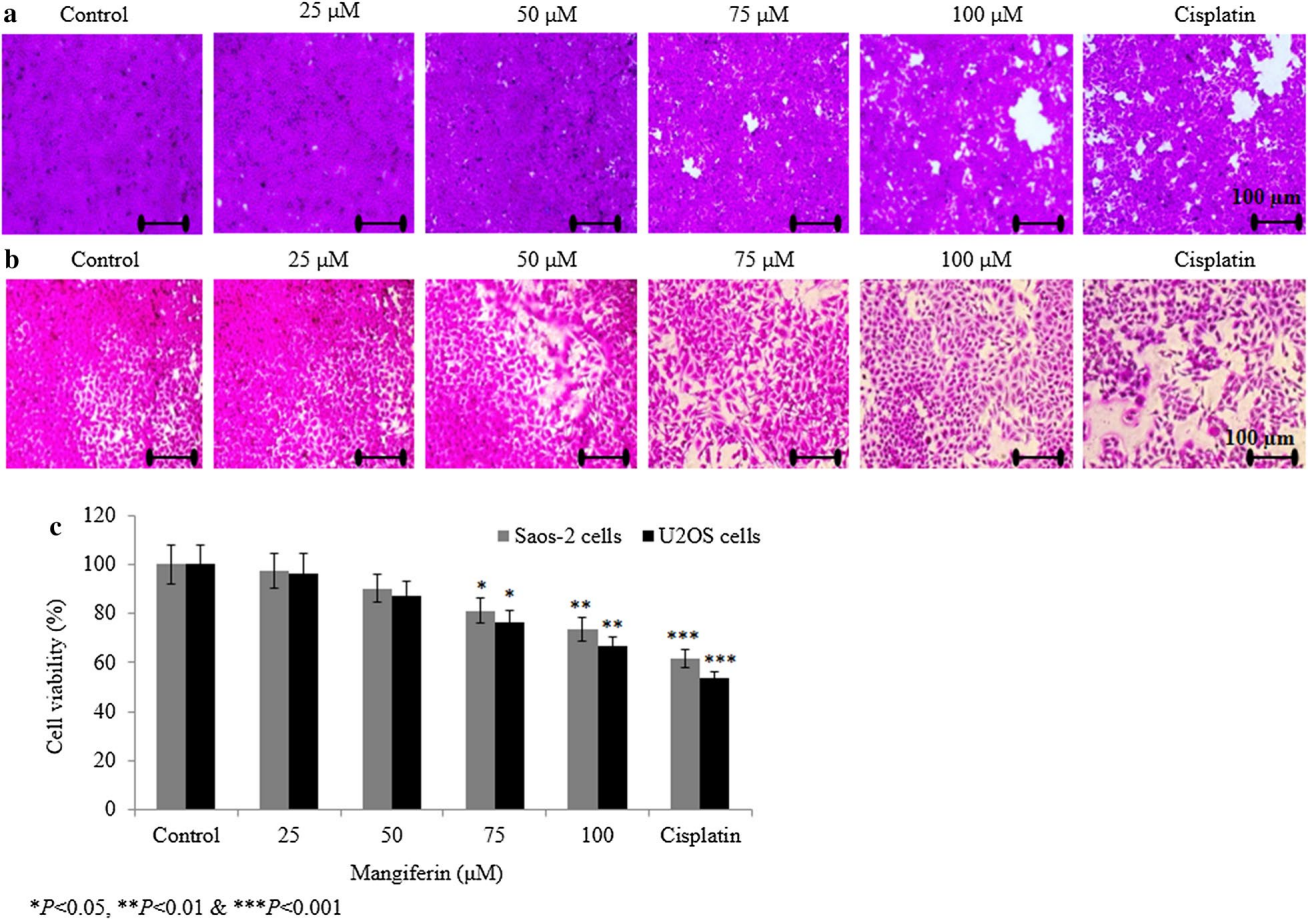
Mangiferin inhibits the cell viability of metastatic osteosarcoma cell. Cancer cells were incubated with mangiferin (25–100 µM) for 72 h. **a** Saos-2 cell viability. **b** U2OS cell viability and **c** percentage analysis of **a** and **b**. Scale bar = 100 µm. N = 6

We investigated the impact of various concentrations of mangiferin on the invasion and proliferation of Saos-2 and U2OS cells following 72 h of treatment. Mangiferin reduced the Saos-2 cell viability to 2.7%, 9.8%, 18.6% and 26.5% at 25, 50, 75 and 100 µM, respectively (*P *< 0.05, Fig. 1a, c). Mangiferin reduced the U2OS cell viability to 3.7%, 12.7%, 23.7% and 33.5% at 25, 50, 75 and 100 µM, respectively (*P *< 0.05, Fig. 1b, c).

### Effect of mangiferin on osteosarcoma cell colony formation

**Fig. 2:**
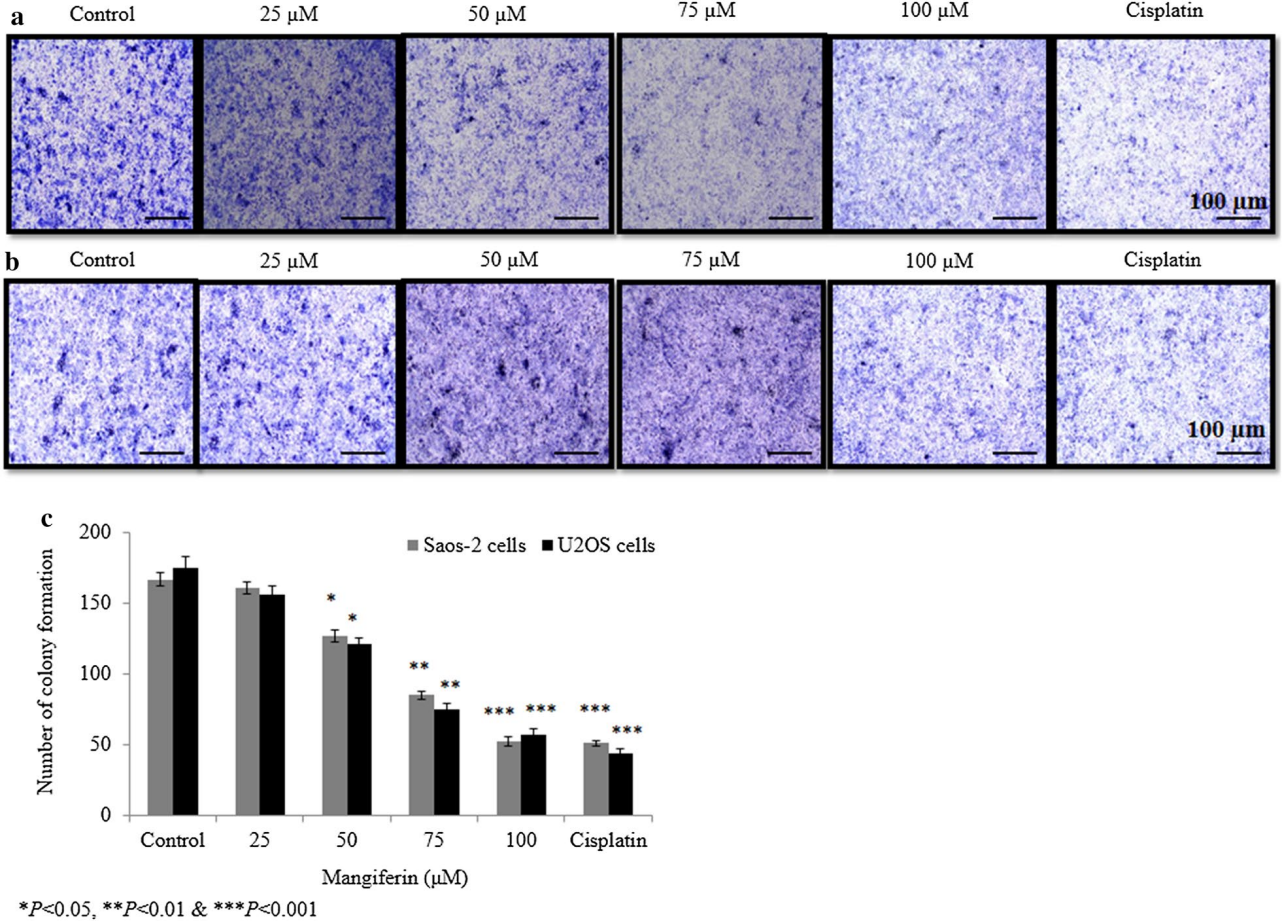
Effect of mangiferin on the ability of osteosarcoma cells to form colonies. Cells were incubated with mangiferin (25–100 µM) for 72 h. **a** Saos-2 cell colony formation assay, **b** U2OS colony formation assay and **c** percentage analysis of a and b. N = 6. Control vs. *P < 0.05. Scale bar is 100 µm

The impact of mangiferin on the ability of cells to form colonies was assessed using clonogenic assays. Mangiferin decreased proliferative potential of Saos-2 cells to 3.5%, 23.9%, 49.1% and 68.9% at 25, 50, 75 and 100 µM, respectively (*P *< 0.05, Fig. 2a, c). Mangiferin decreased the proliferative potential of U2OS cells to 10.8%, 30.8%, 57.1% and 67.4% at 25, 50, 75 and 100 µM, respectively (*P *< 0.05, Fig. 2b, c).

### Effect of mangiferin on apoptosis of osteosarcoma cells

**Fig. 3:**
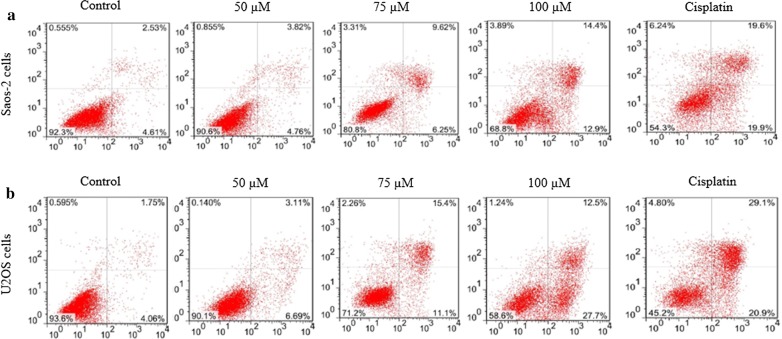
Mangiferin effect on the osteosarcoma cell apoptosis. Cells were incubated with mangiferin (50–100 µM) for 72 h. **a**: represents the Annexin-VFITC of Saos-2 cells, **b**: represents the Annexin-VFITC of U2OS cells. N = 6

Mangiferin-induced cell apoptosis was assessed using Annexin-V/PI staining. A total of 3.82%, 9.62% and 14.4% of Saos-2 cells were non-viable following incubation with 10, 15 and 20 µM mangiferin, respectively (Fig. 3), whereas a total of 3.11%, 12.5% and 15.4% of U2OS cells were non-viable following incubation with 50, 75 and 100 µM mangiferin, respectively (Fig. 3).

### Effect of mangiferin on osteosarcoma cell detachment and cell adhesion

**Fig. 4:**
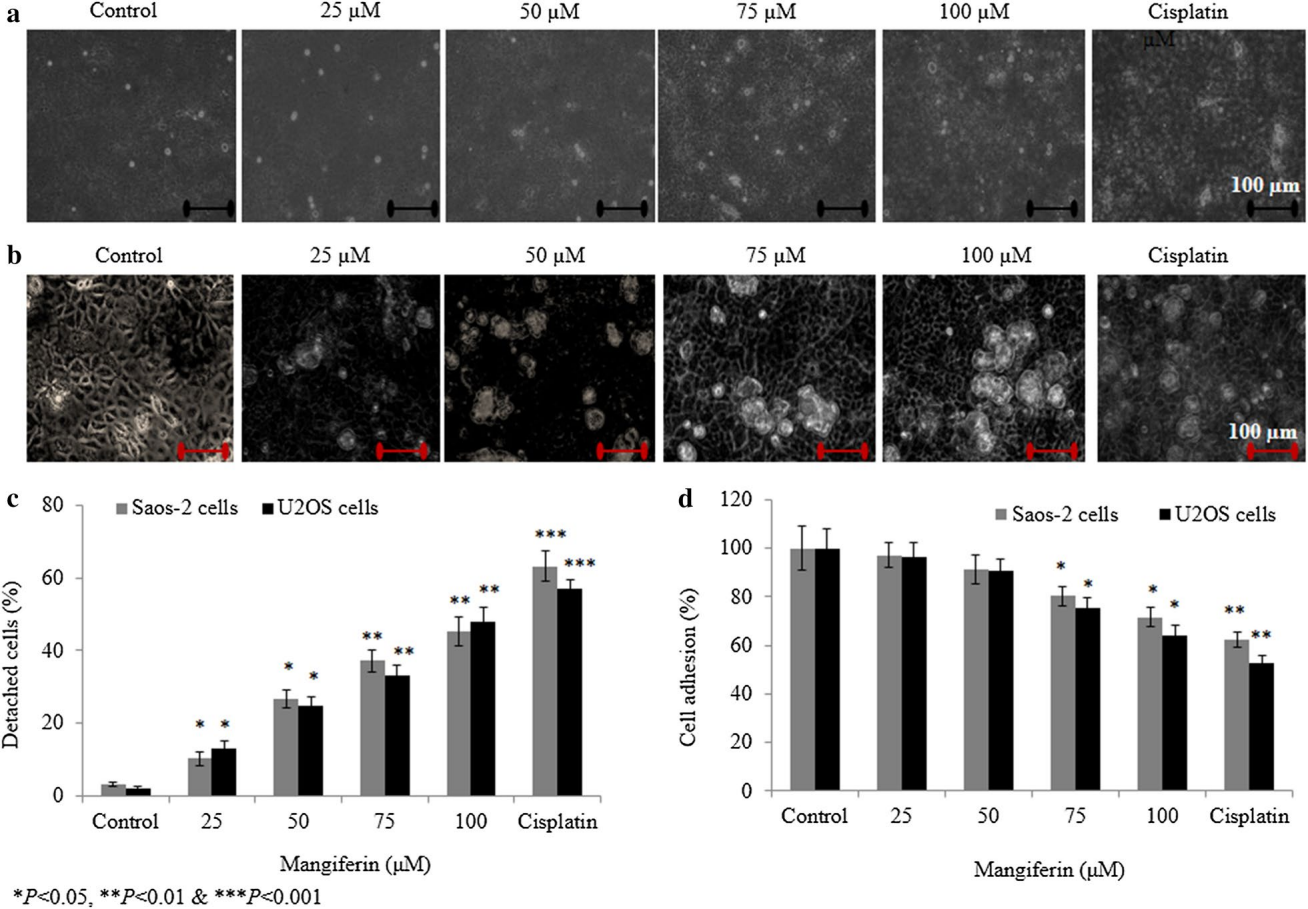
Effect of mangiferin on the osteosarcoma cell detachment and cell adhesion. Cells were incubated with mangiferin (25–100 µM) for 72 h. **a** Represents the detachment level of Saos-2 cells, **b** Represents the detachment level of U2OS cells and **c** percentage analysis of a and b. D: Represents the cell adhesion rate. N = 6. Control vs. *P < 0.05

Mangiferin increased the percent of detached Saos-2 cells to 9.1%, 26.2%, 37.4% and 46.2% at 25, 50, 75 and 100 µM, respectively (Fig. 4a, c), whereas mangiferin increased U2OS cell detachment to 13%, 25%, 33% and 48% at 25, 50, 75 and 100 µM, respectively (Fig. 4b, c). Mangiferin decreased the Saos-2 cell adhesion rate to 2.8%, 8.5%, 19.6% and 28.4% at 25, 50, 75 and 100 µM, respectively (P < 0.05, Fig. 4d), whereas U2OS cell adhesion rate reduced to 3.9%, 9.3%, 24.6% and 35.7% at 25, 50, 75 and 100 µM, respectively (P < 0.05, Fig. 4d).

### Effect of mangiferin on migration rate and invasive potential of osteosarcoma cells

**Fig. 5:**
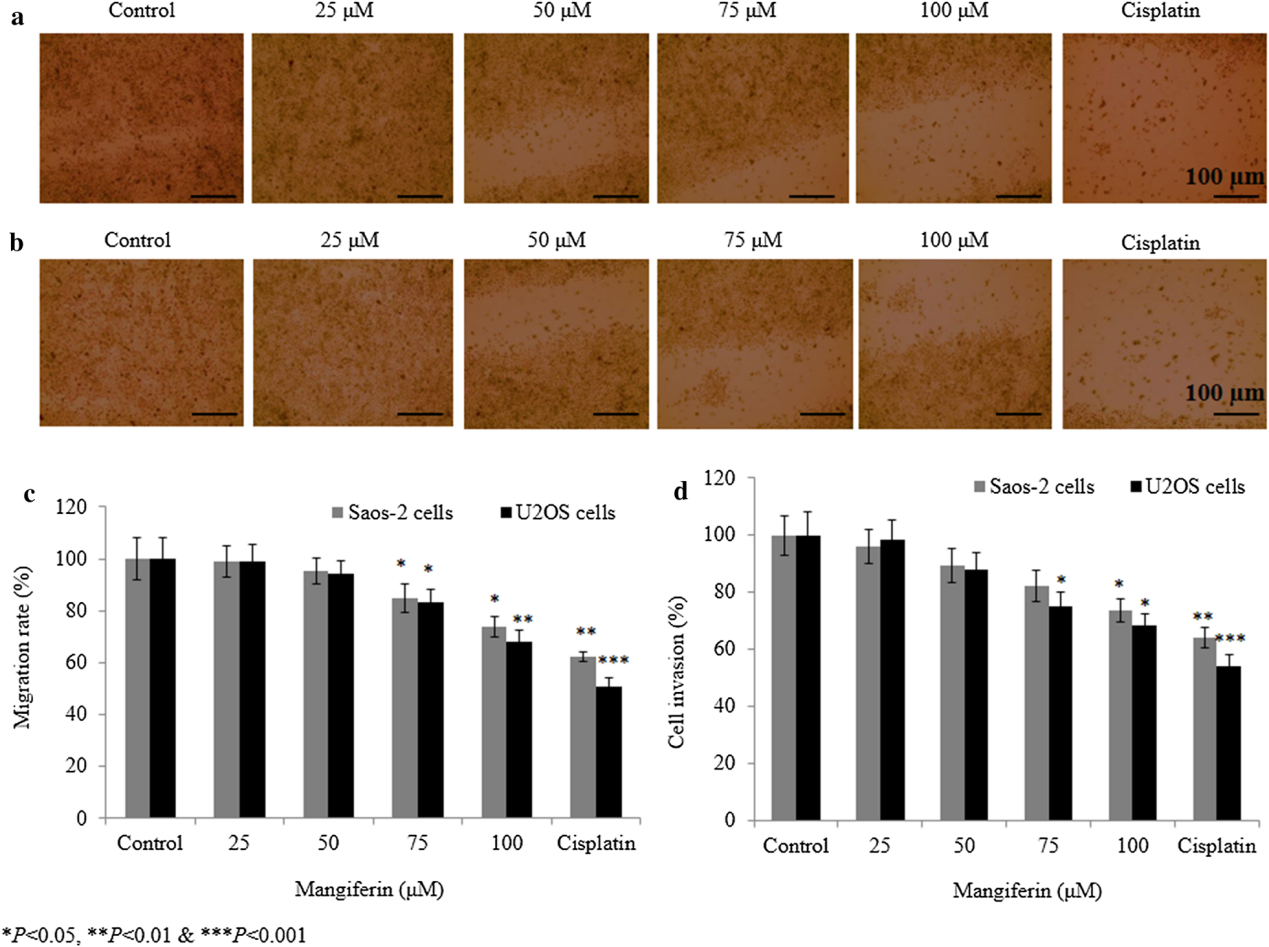
Effect of mangiferin on cell migration and invasion. Cells were incubated with mangiferin (25–100 µM) for 72 h. **a** representative microscopic images of the Saos-2 cell migration assay, **b** representative microscopic images of the U2OS cell migration assay, **c** percent migration rate and **d** percent cell invasion. N = 6. Control vs. *P < 0.05. Scale bar is 100 µm

Mangiferin reduced the Saos-2 cell migration rate to 0.8%, 4.7%, 15% and 26.3% at 25, 50, 75 and 100 µM, respectively (P < 0.05, Fig. 5a, c), whereas mangiferin decreased U2OS cell migration rate to 1.2%, 5.7%, 16.6% and 31.7% and 49% at 25, 50, 75 and 100 µM, respectively (Fig. 5b, c). Mangiferin decreased the invasive potential of Saos-2 cells to 3.8%, 10.6%, 17.8% and 26.3% at 25, 50, 75 and 100 µM, respectively (Fig. 5d), whereas mangiferin decreased U2OS cell invasive potential to 1.7%, 12.2%, 24.7% and 31.7% at 25, 50, 75 and 100 µM, respectively (P < 0.05, Fig. 5d).

### Effect of mangiferin on MMP and TIMP

**Fig. 6:**
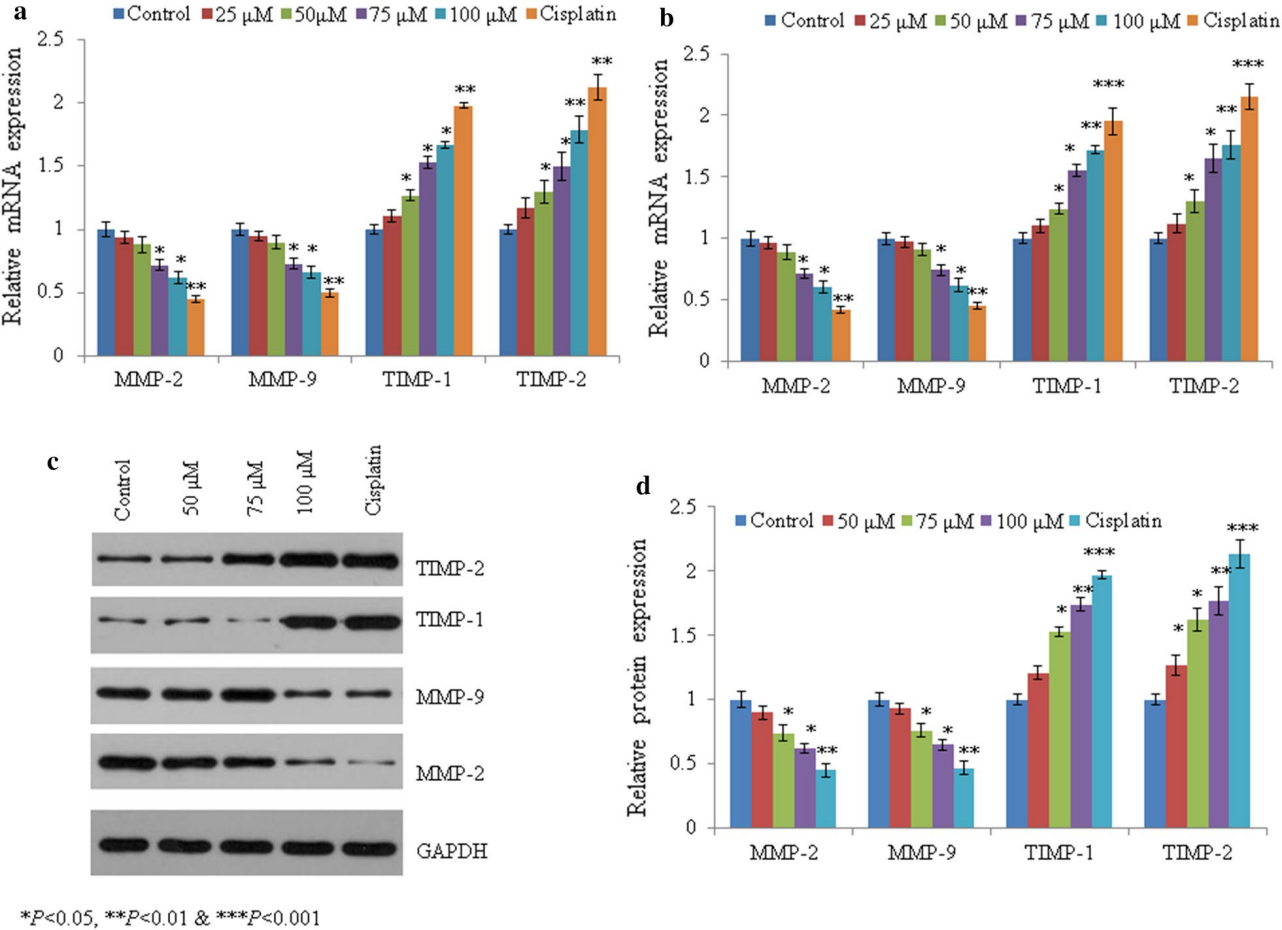
Effect of mangiferin on the mRNA and protein expression of TIMP-1/2 and MMP-2/9 in osteosarcoma cells. Cells were incubated with mangiferin (25–100 µM) for 72 h. **a** Represents the mRNA expression in Saos-2 cells and **b** mRNA expression in U2OS cells. **c** Western blot bands in Saos-2 cells and **d** densitometry analysis of C. N = 6. Control vs. *P < 0.05

In Saos-2 cells, mangiferin decreased mRNA expression of MMP-2 by 0.06-, 0.12-, 0.28 and 0.38-fold, as well as MMP-9 levels by 0.05-, 0.10-, 0.27 and 0.34-fold at 25, 50, 75 and 100 µM, respectively (*P *< 0.05, Figs. 6a). Mangiferin increased mRNA expression of TIMP-1 by 0.11-, 0.27-, 0.53 and 0.67-fold at 25, 50, 75 and 100 µM, respectively. The mRNA expression of TIMP-2 increased by 0.05-, 0.10-, 0.27 and 0.34-fold at the same concentrations (*P *< 0.05, Fig. 6a). Similar effect was noted in U2OS cells following mangiferin (*P *< 0.05, Fig. 6b). Mangiferin showed similar effect on protein like mRNA expression (*P *< 0.05, Fig. 6c, d).

### Effect of mangiferin on expression of PTHR1

**Fig. 7:**
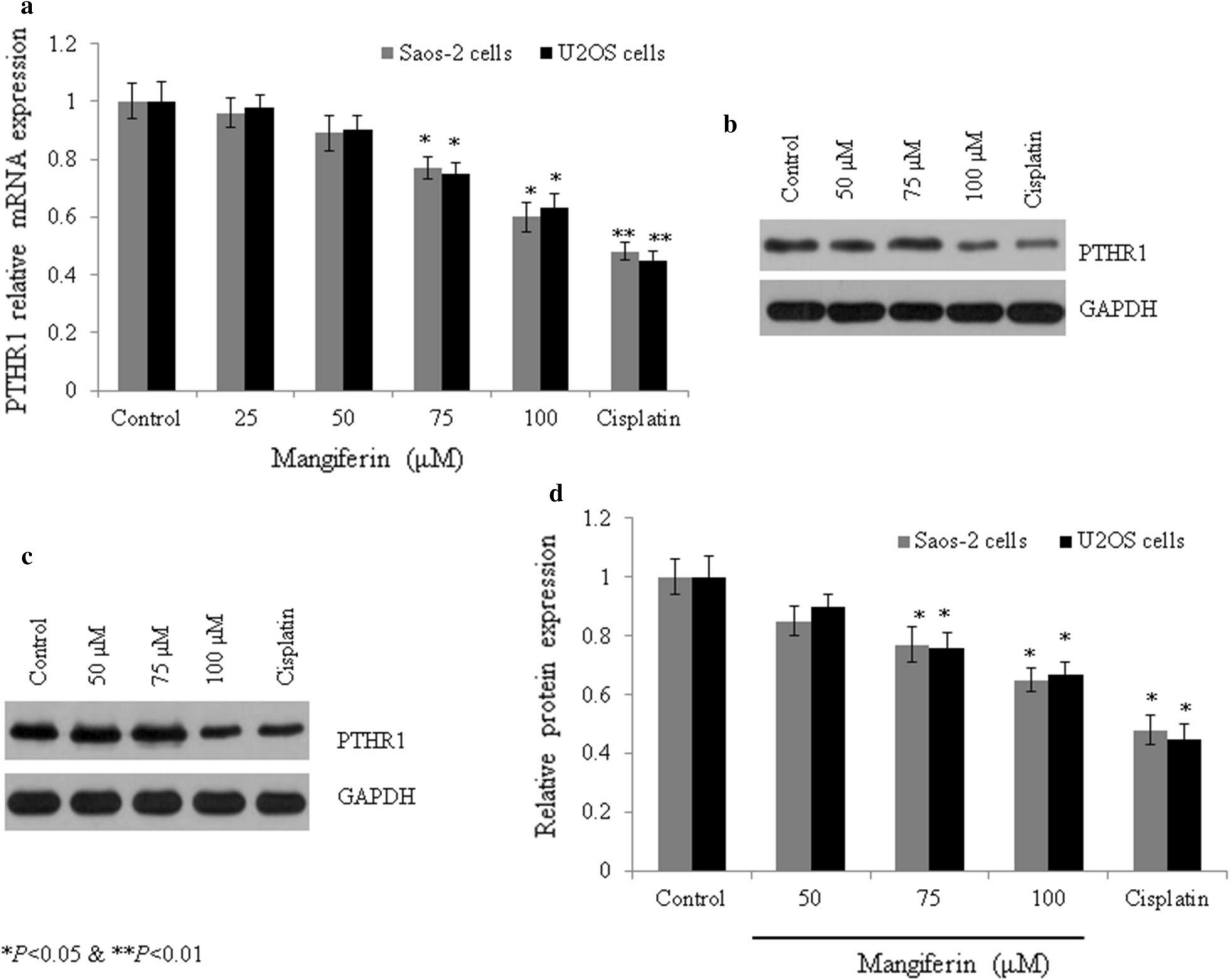
Mangiferin effect on the mRNA and protein expression of PTHR1 in osteosarcoma cells. Cells were incubated with mangiferin (50–100 µM) for 72 h. **a** mRNA expression of PTHR1 in Saos-2 and U2OS cells, **b** western blot bands of PTHR1 in Saos-2 cells, **c** western blot bands of PTHR1 in U2OS cells. **d** Densitometry analysis of **b** and **c**. N = 6. Control vs. *P < 0.05

**Fig. 8:**
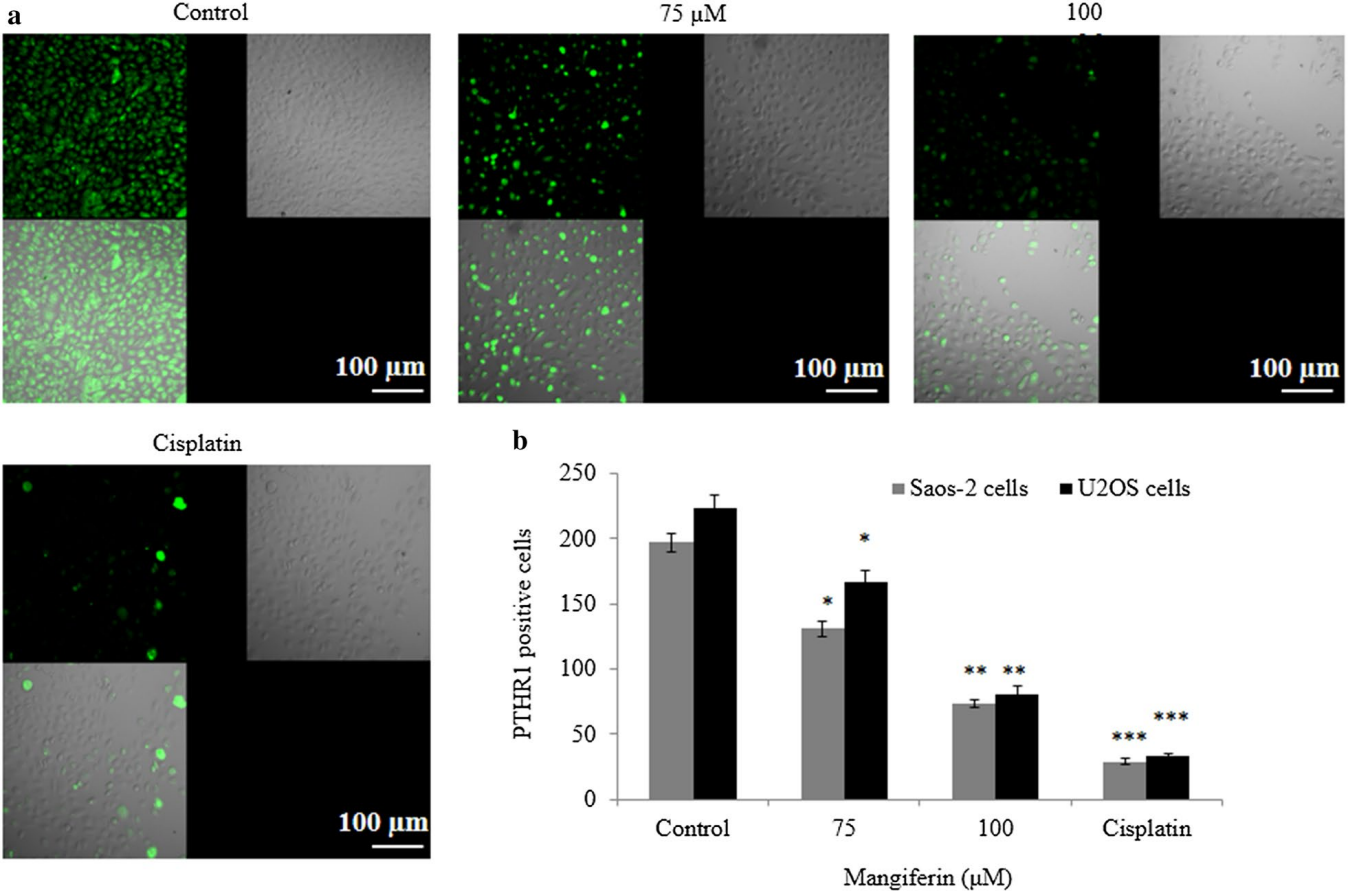
Protein expression of PTHR1, as determined by immunohistochemistry in osteosarcoma cells following mangiferin treatment. Cells were treated with mangiferin (75 and 100 µM) for 72 h. **a** Immunohistochemical confocal images in Saos-2 cells and **b** PTHR1-positive cells. N = 6. Control vs. *P < 0.05. Scale bar is 100 µm

In Saos-2 cells, mangiferin also reduced mRNA expression of PTHR1 by 0.05-, 0.13-, 0.22 and 0.39-fold at 25, 50, 75 and 100 µM, respectively (*P *< 0.05, Fig. 7a). Similar effect was noted in U2OS cells following mangiferin treatment. Mangiferin showed similar effect on protein like mRNA expression (*P *< 0.05, Fig. 7b–d). In Saos-2 cells, immunohistochemical staining showed decreased protein level of PTHR1 following incubation with mangiferin. PTHR1 protein level was decreased 33.5% and 62.9% at 15 and 20 µM, respectively (*P *< 0.05, Fig. 8a, b), whereas in U2OS cells, mangiferin decreased protein expression of PTHR1 25.1% and 63.7% at 75 and 100 µM, respectively (*P *< 0.05, Fig. 8b).

## Discussion

In our study, we showed that the mangiferin inhibited cell proliferation of Saos-2 and U2OS cells. We speculate that the inhibition of proliferation, invasion and migration are associated with the inhibition of PTHR1. In addition, mangiferin treatment inhibited the subcuta-neous osteosarcoma tumor growth and proliferation (data not shown). These results indicate the antitumor activity of mangiferin against osteosarcoma. Tsai et al. (2014) have showed killing effect of paclitaxel against osteosarcoma cells. However, toxic side effects and drug resistance are main disadvantage of paclitaxel which leads to major problem for their clinical application. Mangiferin is well-known xanthone found in several mango fruits such as barks, peel, leaves, stone, stalks and kernel, and in higher plants (Imran et al. 2017). Dar et al. (2005) have reported the several pharmacological effects of mangiferin such as antioxidant, anticancer, antiaging, antiviral, hepatoprotective, analgesic and immunomodulatory potential. An acidic environment is mandatory for tumor cell proliferation and invasion (Rofstad et al. 2006). In the current study, the reduced adhesion, cell proliferation, invasion, and migration confirmed that tumor cell metastasis occurred following treatment with mangiferin.

Do Thi et al. (2014) reported that degradation of the extracellular matrix (ECM) is a key step in metastasis. Matrix metalloproteases (MMPs) play key role in degradation of the ECM, which leads to increased acceleration and migration of metastatic tumor (Libra et al. 2009). Guo et al. (2015) also reported that the inhibition of invasive and migratory potential of A549 cells through reduced expression of MMP–2/9. In this study, MMP-2/9 mRNA and protein expression was reduced significantly following treatment with mangiferin. These results indicate that the anti-metastatic effect of mangiferin. We speculate that the inhibition of MMP-2/9 is key mechanism for the inhibition of osteosarcoma. Therefore, the inhibition of PTHR1 expression could be useful therapeutic approach for the treatment of osteosarcoma. In this study, mangiferin reduced the mRNA and protein expression of PTHR1, which further confirms the anti-metastatic effect of mangiferin. In summary, we have demonstrated that treatment with mangiferin reduces cell viability, invasion, adhesion, proliferation, and migration, and induces apoptosis osteosarcoma cells. Therefore, treatment with mangiferin can be effective agent in inhibiting growth and inducing apoptosis in osteosarcoma cells. Our experimental data provide evidence for the therapeutic effect of mangiferin in osteosarcoma cells.

## Data Availability

Corresponding author could provide the all experimental data on valid request.
